# Collaboration at Communications Biology

**DOI:** 10.1038/s42003-019-0632-y

**Published:** 2019-10-11

## Abstract

Our editorial model is a collaborative one; the in-house professional team of editors works together with external editors in selecting the papers we publish. Here we discuss how this editorial model is set up and share the thoughts of some of our board members.

The editorial team at *Communications Biology* is a mix of in-house professional editors and external Editorial Board Members (EBMs). Whereas all of the professional editors at the journal have a research background, our external editors are actively conducting research and, as such, bring additional technical expertise as well as knowledge of the current state of their research area. Working very similarly to the in-house team, our EBMs handle papers from the initial submission up to the formal acceptance stage.

We strive to be clear and transparent in our editorial decisions so that, even if the outcome is not positive, authors should understand the reason for the rejection.

Our dedicated EBMs (there are currently 20) work in diverse fields of biology, but together with the in-house editors, they share the wish to provide the best possible service to our authors. We do try to be fast, but speed is not at the expense of rigour and thoughtfulness in our assessments. For a majority of the papers, the handling editor discusses the paper with at least one other member of the editorial team. This holds true for papers handled both by in-house and external editors. Such discussions are held throughout the peer review process and are essential in order for us to share editorial and scientific expertise and to be consistent in our decision making. See https://www.nature.com/commsbio/journal-policies/editorial-process for more on our editorial processes.

Open communication is key goal for us. We strive to be clear and transparent in our editorial decisions so that, even if the outcome is not positive, authors should understand the reason for the rejection. Equally important is providing guidance when inviting our authors to revise a paper following peer review. The editors will specify what referee points are the most essential ones to address and may overrule on requests that appear beyond the scope of the study.

Many of our EBMs are at a relatively early stage of their careers as independent researchers and feel that taking part in editorial work gives them some useful insights. We asked four of them about their experience so far and their feedback was similar and reassuringly positive. Our expert in macro-ecology and evolution, Katie Davis (https://drkatiedavis.wordpress.com/), finds that she has learnt a lot about the editorial process. “I’m a lot more understanding and patient with my own manuscripts now that I know just how hard editors have to work,” she remarks. “It’s informative to see the other side of the submission process and how the mechanics work,” agrees Ngan Huang (http://med.stanford.edu/huanglab.html), who handles papers in biomedical engineering and stem cells. Georgios Giamas (http://www.sussex.ac.uk/lifesci/giamaslab/) notes, “I am (or have been) part of different editorial boards. However, in this case, there is more interaction and discussion with the in-house editorial team, which gives me the opportunity to see some things from a different perspective”.

Although *Communications Biology* will not decline a manuscript because of how it is written, the external editors we talked to also share the realisation that presenting their findings in a clear manner is helpful. Si Ming Man (https://jcsmr.anu.edu.au/people/academics/dr-si-ming-man) remarks: “I have learnt that I have to tell the editor what is important and put together a well-written manuscript.” Indeed, clarity in presentation helps the editor, and more importantly, makes the review process smother and helps readers understand the importance of the work.

*Communications Biology* aims to provide a home for papers in a wide range of biological research areas. Therefore, just like the in-house editors, our EBMs sometimes have to handle papers somewhat outside of their own research focus, which initially can be a challenge. “This was daunting at first,” admits Si Ming, who is in the field of innate immunity. “But,” he continues, “I realised that you do not need to be an expert but you have to invest more time to understand the contribution.” The four EBMs we spoke to here also appreciate the opportunity to learn about science outside of their immediate research areas.

Finally, our EBMs are so much more than manuscript editors. They are in a great position to actively engage with their research communities as ambassadors for *Communications Biology*. As a new journal that is still fighting to be a “household name,” we are very grateful for their efforts in promoting the journal among their colleagues. They can also provide feedback and suggestions on editorial processes and journal content and constitute a sounding board for testing new ideas.

We are privileged to work with our group of external editors, and we look forward to develop our collaboration further as the journal grows and evolves.

